# Prevalence and risk factors associated with *Clonorchis sinensis* infections in rural communities in northern Vietnam

**DOI:** 10.1371/journal.pntd.0008483

**Published:** 2020-08-03

**Authors:** Thao T. B. Nguyen, Veronique Dermauw, Hafid Dahma, Dung Thi Bui, Trang T. H. Le, Ngan T. T. Phi, Laetitia Lempereur, Bertrand Losson, Olivier Vandenberg, Dung Trung Do, Pierre Dorny

**Affiliations:** 1 School of public health, Université Libre de Bruxelles (ULB), Brussels, Belgium; 2 Center for Public health and Ecosystem Research, Hanoi University of Public Health, Hanoi, Vietnam; 3 Department of Biomedical Sciences, Institute of Tropical Medicine Antwerp, Belgium; 4 Department of Microbiology, LHUB—ULB, Groupement Hospitalier Universitaire de Bruxelles (GHUB), Université Libre de Bruxelles (ULB), Brussels, Belgium; 5 Department of Parasitology, Institute of Ecology and Biological Resources, Vietnam Academy of Science and Technology, Hanoi, Vietnam; 6 Faculty of Ecology and Biological Resources, Graduate University of Science and Technology, Vietnam Academy of Science and Technology, Hanoi, Vietnam; 7 Faculty of Veterinary Medicine, Kasetsart University, Bangkok, Thailand; 8 Faculty of Science and Technology, University of Twente, Enschede, Netherlands; 9 Laboratory of Parasitology and Parasitic Diseases, Faculty of Veterinary Medicine, Université de Liège, Liège, Belgium; 10 Center for Environmental Health and Occupational Health, School of Public Health, Université Libre de Bruxelles (ULB), Brussels, Belgium; 11 Innovation and Business Development Unit, LHUB—ULB, Groupement Hospitalier Universitaire de Bruxelles (GHUB), Université Libre de Bruxelles (ULB), Brussels, Belgium; 12 Division of Infection and Immunity, Faculty of Medical Sciences, University College London, London, United Kingdom; 13 Department of Parasitology, National Institute of Malariology, Parasitology and Entomology, Hanoi, Vietnam; Seoul National University College of Medicine, REPUBLIC OF KOREA

## Abstract

**Background:**

Clonorchiasis, caused by the fish-borne trematode *Clonorchis sinensis*, is a neglected tropical disease and a public health issue in endemic countries. In Vietnam, an in-depth analysis of risk factors for the condition is missing up to now. This study aimed to determine the prevalence of *C*. *sinensis* infection and associated risk factors in rural communities in northern Vietnam.

**Methodology/Principal findings:**

A cross-sectional survey was conducted in 4 communes in Yen Bai and Thanh Hoa provinces where clonorchiasis is known to be present and raw fish consumption is a common. Using a simple random sampling approach, stool was collected from 841 participants over 6 years old for coprological examination, and a questionnaire measured knowledge, attitudes, and practices with regard to clonorchiasis in 757 participants over 15 years old. Univariable and multivariable logistic regression models were run to identify risk factors for infection with *C*. *sinensis*. The overall prevalence of *C*. *sinensis* infection was 40.4%, with commune prevalences ranging between 26.5% and 53.3%. In the final model, males were significantly more likely to be infected with *C*. *sinensis* (OR 2.00; 95% CI 1.31–3.05). Recent (i.e. last year) consumption of raw fish (OR 8.00, 95% CI 4.78–13.36), low education level (OR 5.57; 95% CI 2.37–13.07), lack of treatment (OR 1.82, 95% CI 1.15–2.89), being between 19 to 39 years old (OR 6.46; 95% CI 1.25–33.37), and the presence of an unhygienic toilet (OR 2.74, 95% CI 1.53–4.92) were significantly associated with *C*. *sinensis* infection.

**Conclusion/Significance:**

This study demonstrated a high prevalence of *C*. *sinensis* infection in rural communities in northern Vietnam. Thus, control measures including, mass drug administration for those communes should be applied to reduce the prevalence. Moreover, specific health education activities should be developed for risk groups in *C*. *sinensis* endemic areas.

## Author summary

Clonorchiasis, caused by the fish-borne trematode *Clonorchis sinensis*, is a chronic liver infection and is classified as a neglected tropical disease, particularly in some Asian countries such as Vietnam. Light infections with *C*. *sinensis* are asymptomatic, yet heavy chronic infections are associated with clinical complications such as, bile duct obstruction, hepatic fibrosis and the most serious complication being bile duct cancer. We carried out a community-based study on *C*. *sinensis* infection in rural communities in northern Vietnam. Our results indicated that the Thac Ba lake area in Yen Bai province is a hot spot of *C*. *sinensis* infection, and that Thanh Hoa province remains an area with widespread small liver fluke infection. Eating raw fish was confirmed to be an important risk factor for *C*. *sinensis* infection in Vietnam. Presence of infection was furthermore associated with gender, age, education and hygienic practices. Mass drug administration and improved awareness campaigns for the population in these regions are needed. Setting-specific interventions targeting different risk groups should be considered to reduce the disease transmission.

## Introduction

Clonorchiasis is a neglected tropical disease caused by the liver fluke *Clonorchis sinensis* [1, 2]. *C*. *sinensis* is endemic in China, Taiwan, Korea, eastern Russia and north Vietnam [2, 3] and may spread to non-endemic regions due to the expansion of international markets and increased migration and travelling [3, 4]. The complex life cycle of *C*. *sinensis* requires three host species, a freshwater snail as the first intermediate host, in which the parasites multiply, a freshwater fish of the family Cyprinidae as the second intermediate host, in which the infective larval stages (metacercaria) encyst, and humans and other piscivorous mammals (e.g., cat, dog, pig) as the definitive hosts, in which the adult worms develop [4, 5].

People become infected with *C*. *sinensis* upon consumption of raw, undercooked or salt-preserved fish containing metacercariae [6]. After ingestion, the metacercariae excyst in the duodenum, migrate to the intrahepatic bile duct and develop into adult worms within 16–25 days and can live there for up to 30 years [7–9]. While light infections are asymptomatic, heavy chronic infections are associated with clinical complications such as bile duct obstruction, stone formation, cholangitis, hepatic fibrosis and the most serious complication, cholangiocarcinoma–bile duct cancer [7, 10, 11]. The global number of people currently at risk for *C*. *sinensis* is estimated at 600 million with over 35 million people being infected and 2 million showing symptoms, contributing to more than 5500 deaths each year [2, 7, 12, 13]. The International Agency for Research on Cancer of the WHO classified *C*. *sinensis* as a group I carcinogen in humans [10].

In Vietnam, *C*. *sinensis* is present in the north, while *Opisthorchis viverrini*, the Southeast Asian liver fluke is prevalent in the south and the center of the country [14]. It was estimated by WHO that approximately 1 million people are infected with small liver flukes in Vietnam [14–17]. Reported prevalence of *C*. *sinensis* infections range between 0.2% and 37.5%, with the highest rates found in the Nam Dinh (37.5%) and Ninh Binh (31%) provinces [16, 17]. Few studies have investigated risk factors associated with *C*. *sinensis* infection in Vietnam. One study, conducted in Thanh Hoa province (reported prevalence: 17.2%), established that family size was significantly associated with infection (univariable analysis of social factors) [18]. Dang et al. reporting a prevalence of 26.1% in Ninh Binh province, found that infection was significantly associated with raw fish consumption (univariable analysis, no other factors investigated) [19]. In a more recent cross-sectional study in Ninh Binh province (reported prevalence 16.5%), *C*. *sinensis* infection was significantly associated with raw or undercooked fish consumption, as well as with gender, education and village location (all univariable analyses) [20].

Up to now, few studies have investigated risk factors for clonorchiasis in the region, and an in-depth analysis in Vietnam, using multivariable models, is lacking. In Vietnam, past government campaigns, advising local communities to stop raw fish consumption, have been implemented in some provinces, yet they have not resulted in a sustainable reduction in the prevalence of fish-borne trematode infections, including clonorchiasis [19, 21, 22]. Indeed, in order to achieve successful control of clonorchiasis, it is essential to identify high endemic areas and unravel local risk factors. As behavioural factors related to the disease can differ between regions and countries, it is important to investigate risk factors for clonorchiasis in the Vietnamese setting. This knowledge can subsequently guide the development of efficient control programs, which include health education campaigns.

Therefore, the aims of the current study were i) to estimate the prevalence of *C*. *sinensis* infection and ii) to identify associated risk factors, in four communes in northern Vietnam.

## Methods

### Study sites

**Fig 1:**
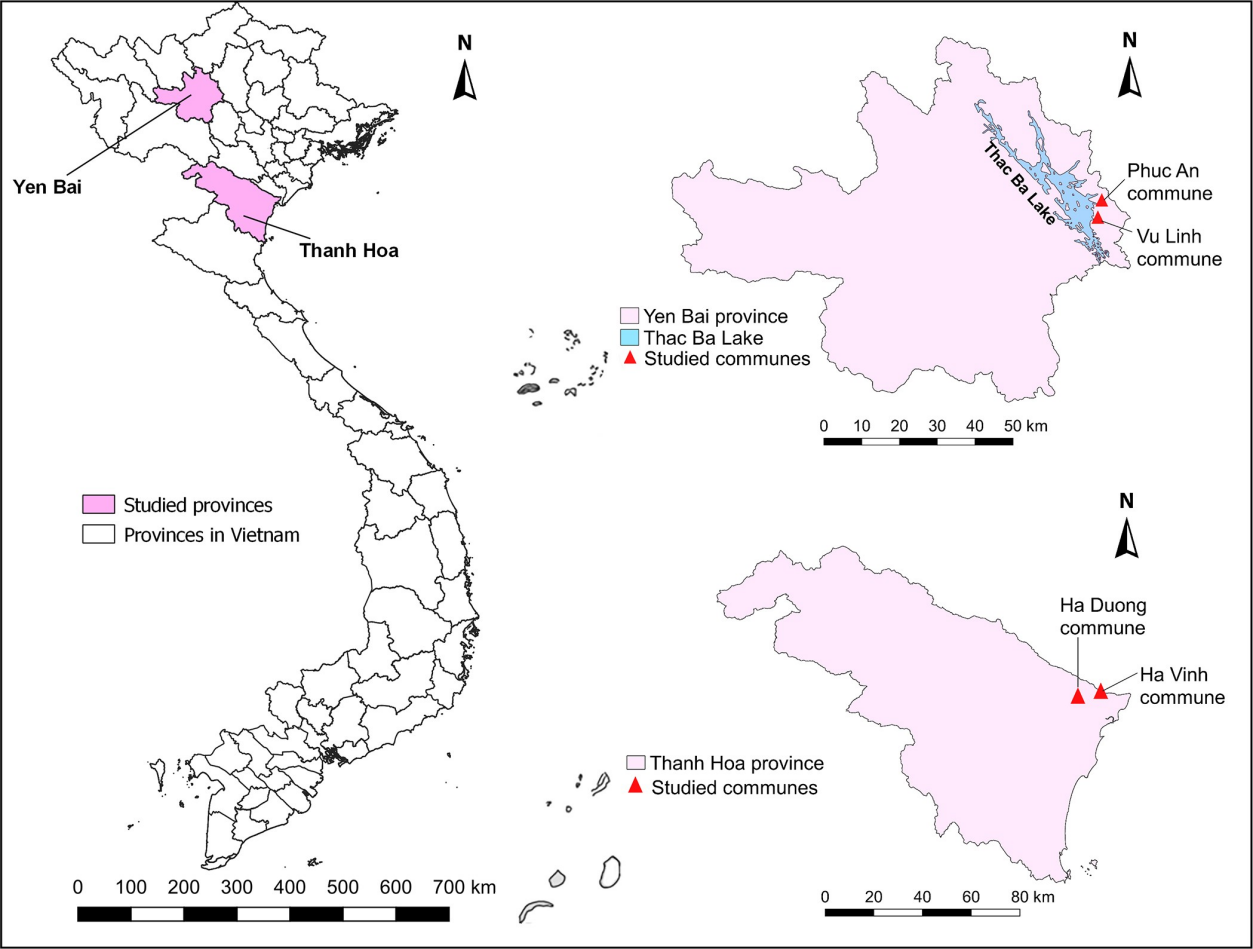
Map of Vietnam showing the locations of Yen Bai and Thanh Hoa provinces and the respective study communes. The map was created using QGIS Desktop version 3.4.11 software.

The study was conducted in Yen Bai and Thanh Hoa provinces (Fig 1), northern Vietnam. We selected these two provinces because recent studies suggested a high endemicity of clonorchiasis: a prevalence of 17% was reported in a commune in Thanh Hoa [18], a coastal province, and more than 36% in a mountainous area of Yen Bai province [23]. Moreover, in these two provinces, raw fish consumption is common [18, 24].

In each province, two communes were selected, after in-depth discussions with staff of provincial and district health centers. In Yen Bai, the communes Phuc An and Vu Linh were selected on the basis of their location close to the Thac Ba lake, one of the biggest artificial lakes in Vietnam, and the main source of fish in the province. Bui et al. [24] recorded a high prevalence (31.1–76.7%) of *C*. *sinensis* infection in four wild-caught fish species originating from the lake. The consumption of raw fish in both communes, bordering the eastern part of Thac Ba lake, is common. Phuc An has a population of 3650 living in 787 households, while those numbers in Vu Linh communes were 5679 and 1272, respectively. Based on interviews with local health workers, there are many clonorchiasis cases in this area. In Thanh Hoa, the communes Ha Vinh and Ha Duong were selected. The selection was based on a high proportion of inhabitants in these communes owning fish ponds, the habit of using fresh human/animal feces as food for fish and the habit of eating raw fish (personal communication, district health center). In addition, the rivers and ditches flowing through the two communes form an important source of fish for the local population. Ha Vinh has a population of 7620 living in 1690 households, while those numbers in Ha Duong commune were 3505 and 761.

### Study design, participant selection

A cross-sectional study was conducted between March and May, 2018. The required sample size for each commune was calculated using the formula for a single population proportion: $n=\frac{z_{\left(1-\alpha /2\right)}^{2}∙p∙(1-p)}{d^{2}}$ [25] with an envisaged confidence interval of 95%, precision of 6% and an estimated infection prevalence of 22%, resulting in a sample size of 183 people per commune (732 people in total). Taking into account failure to submit a stool sample for an estimated 30% of participants, 238 people were invited in each commune.

A simple random sampling approach was used to enroll participants. For each commune, the local community health station was asked to provide a list of households, from which a random subset of households was selected. Community health workers visited the selected household to explain study aims and study procedures. Then, 2 eligible, randomly selected people per household, meeting the inclusion criterion (being above 6 years old) were invited to participate in the study, and provide a stool sample. Participants above 15 years old were additionally invited for an interview.

### Participant procedures

Each enrolled, consenting individual received a labeled sample bag, and was asked to provide a stool sample after receiving guidance from a community health worker on how to collect the sample. Participants returned their stool samples to the community health station on the next or next two days. Sex and age were noted for each participant.

Each participant of 15 years or older was asked to complete a questionnaire after providing the stool sample. An interviewer-assisted questionnaire was conducted using a smartphone with Kobo Toolbox software. The questionnaire consisted of 4 parts: i) socio-demographic information, i.e. age, sex, ethnic group, education, occupation, the presence of a fish pond and household animals, and questions on ii) knowledge, iii) attitude, and iv) practices, with regard to clonorchiasis. The second part was a set of 12 questions related to knowledge on transmission, prevention and symptoms of clonorchiasis (See supplement S1 Table), while the third part consisted of 3 questions, being perceived seriousness (4 levels of seriousness), health concern of clonorchiasis (3 levels of concern) and the desire to understand the disease (yes, no). The fourth part was comprised of a set of 5 questions, on behavioral factors and past experience, being raw fish consumption (never ate raw fish, ate raw fish more than 1 year ago, ate raw fish during the last year; the 3 categories were based on the reported moment of last raw fish consumption), utensil use for raw and cooked food (using the same knife/cutting board or not), history of clonorchiasis examination (yes/no), history of clonorchiasis treatment (yes/no), and type of toilet. People who reported having eaten raw fish in the last year were asked more information related to the species of raw fish, reasons for raw fish consumption, and source of raw fish dishes.

### Stool examination

At the health station, aliquots of stool samples were examined by Kato-Kazt thick smear technique under a light microscope by three experienced technicians to determine the intensity of trematode and other helminth infections (results will be reported elsewhere). About 2–3 g of the remaining stool sample was preserved in 10% of formalin and transferred to the parasitology laboratory of the National institute of Malariology, Parasitology and Entomology (NIMPE) in Hanoi for examination by the formalin–ether concentration technique (FECT).

The FECT was used to identify *C*. *sinensis* eggs in stool as described elsewhere [26]. In brief, the formalin preserved fecal sample was homogenised, and then 2–3 ml of the stool suspension was added to 10 ml of a 10% formalin solution. The tube was mixed thoroughly and filtered through a funnel with gauze into new tube. Next the filtered solution was added to a 10% formalin solution until a volume of 7 ml was reached, and 3 ml of diethyl ether was added, the solution was then mixed thoroughly during 10 seconds before being centrifuged for 3 minutes at 500 *g*. The sediment was mixed with normal saline and examined for *C*. *sinensis* eggs under a microscope at 10 × and 40 ×. Eggs of *C*. *sinensis* were distinguished from those of minute intestinal fluke (MIF) based on morphological characteristics: primary criteria for *C*. *sinensis* egg identification were the oval shape and a rough skin and muskmelon patterns on the egg shell surface [27, 28]; in case of a smooth surface, the prominent margin of the operculum appearing as shoulder rims, and knob were considered as the secondary criteria [27].

### Data analysis

The stool examination and questionnaire data were entered in an Excel file and cross-checked by two independent researchers. A knowledge score was given for participants responding to the questionnaire. A correct answer scored “1” while an incorrect answer scored “0”. The scores for each of the 12 knowledge questions were then summed, and the final score was recoded as follows: i) low: below or equal to 6, ii) medium: 7 to 9 points, and iii) high: above 10 points. An in-depth analysis of specific questionnaire responses for the knowledge questions will be reported elsewhere. The toilet type was categorized in 3 groups: i) hygienic toilet: dry latrine single vault, dry latrine double vault, septic tank, ii) unhygienic toilet: pour flush, temporary pit, “*cau tom*”- a kind of overhung latrine consisting of two dried bamboo sticks hanging above a pond, allowing direct defecation into the pond, and iii) no toilet.

Descriptive statistics were used to describe the prevalence of *C*. *sinensis* infection and questions related to fish eating behavior. Chi-squared tests were used to compare the proportion of raw fish consumption by gender, and age, and compared the resources of fish and the species of fish for raw fish dishes preparation between the two provinces. Subsequently, univariable logistic regression analyses, with and without adjustment for commune, were run to determine the association between *C*. *sinensis* infection and questionnaire responses. Factors for which the univariable logistic regression models had a *p*-value ≤ 0.1 were included in a multivariable logistic regression model. A backward variable selection method was used to determine the most parsimonious model. Model fit was evaluated by means of likelihood ratio testing. The statistical significance level was set a 5%. The data were analyzed using STATA version 12.0 for Windows.

### Ethical approval

The study was approved by the Science, Technology and Ethics committee of NIMPE, number 113/QD-VSR, January 25^th^, 2018, Vietnamese Ministry of Health. Prior to the field work, the provincial and district health officers were informed about the study aims and procedures, and authorization was obtained. Detailed information on study objectives and procedures was provided to each participant, and a written informed consent was obtained prior to enrolment. Children between 6–18 years old were asked for verbal assent and their parents were invited to sign an informed parental consent form. All egg-positive individuals enrolled in the study were treated free of charge with drugs according to the treatment protocol of the Vietnamese Ministry of Health.

## Results

### Characteristic of the study population

A total of 841 individuals participated in the study. The majority was female (446 (53%)), and the mean age was 42 years (range: 6–77, standard deviation (SD) = 14.7). Of these, 404 (48%) were from Thanh Hoa province and 437 (52%) from Yen Bai province. In Thanh Hoa, participants predominantly belonged to the Kinh ethnic group (98.6–100%), while in Yen Bai, participants belonged mainly to Dao ethnic groups (43.0–53.3%) and Kinh (33.8–43.0%) (Table 1). The majority of the study participants were farmer (56.8%), and most finished secondary school (43.7%). Of the 841 participants, 52 children were below 15 years old (6.2%), and 32 people did not participate or complete the questionnaire (3.8%). Hence, 757 people completed the questionnaire, their data were retained for the multivariable analysis.

**Table 1:** Demographic information of the study population.

	Commune (No. sampled;%)				
	Thanh Hoa province (N = 404)		Yen Bai province (N = 437)		Overall
	Ha Duong (N = 185;22.0)	Ha Vinh (N = 219;26.0)	Phuc An (N = 226;26.9)	Vu Linh (N = 211;25.1)	
** Gender (N = 841)**					
Male	99 (53.5)	109 (49.8)	100 (44.3)	87 (41.2)	395 (47.0)
Female	86 (46.5)	110 (50.2)	126 (55.7)	124 (58.8)	446 (53.0)
**Age (years) (N = 841)**					
≤18	22 (11.9)	1 (0.5)	24 (10.6)	29 (13.7)	76 (9.0)
19 to 39	35 (18.9)	48 (21.9)	107 (47.4)	63 (29.9)	253 (30.1)
40 to 59	99 (53.5)	140 (63.9)	88 (38.9)	97 (46.0)	424 (50.4)
≥60	29 (15.7)	30 (13.7)	7 (3.1)	22 (10.4)	88 (10.5)
** Ethnic group (N = 757)**					
Kinh	165 (100.0)	215 (98.6)	66 (33.8)	77 (43.0)	523 (69.1)
Tay	0 (0.0)	0 (0.0)	4 (2.1)	19 (10.6)	23 (3.0)
Dao	0 (0.0)	0 (0.0)	104 (53.3)	77 (43.0)	181 (23.9)
Cao Lan	0 (0.0)	0 (0.0)	20 (10.3)	4 (2.2)	24 (3.2)
Other	0 (0.0)	3 (1.4)	1 (0.5)	2 (1.1)	6 (0.8)
** Education (N = 757)**					
Primary school or no education	23 (13.9)	61 (28.0)	80 (41.0)	64 (35.8)	228 (30.1)
Secondary school	79 (47.9)	106 (48.6)	70 (35.9)	76 (42.5)	331 (43.7)
High school	46 (27.9)	37 (17.0)	33 (16.9)	29 (16.2)	145 (19.2)
Vocational & bachelor	17 (10.3)	14 (6.4)	12 (6.2)	10 (5.6)	53 (7.0)
** Occupation (N = 757)**					
Famer	86 (52.1)	117 (53.7)	112 (57.4)	115 (64.3)	430 (56.8)
Government service & worker	43 (26.1)	28 (12.8)	21 (10.8)	15 (8.4)	107 (14.1)
self-employed	17 (10.3)	47 (21.6)	41 (21.0)	38 (21.2)	143 (18.9)
Student	12 (7.3)	2 (0.9)	2 (1.0)	3 (1.7)	19 (2.5)
Trade	1 (0.6)	12 (5.5)	7 (3.6)	8 (4.5)	28 (3.7)
Mason & fisherman	6 (3.6)	12 (5.5)	12 (6.2)	0 (0.0)	30 (4.0)

### Knowledge, attitude, and practices

A number of participants (17.3%) reported that they had never heard about liver fluke infection. Among people who had heard about clonorchiasis, a high proportion of participants knew that raw fish consumption can lead to infection with the parasite (67%). However, a number of participants had misconceptions about disease prevention: e.g. 7.1% indicated that alcohol drinking when combined with eating raw fish could kill the parasites, while 8.3% mentioned that adding lemon and chili to raw fish would effectively kill the parasites. Moreover, 16.3% indicated that people cannot become re-infected, and 26.4% thought that clonorchiasis can transmit directly between people. Overall, 12.3% of participants had a high level, 50.2% had a medium level, and 37.5% had a low level of knowledge on clonorchiasis.

The vast majority of participants (76.4%) considered clonorchiasis as a serious or very serious disease. Approximately the same number of participants (79.9%) indicated that they were concerned about this disease, while 97.2% mentioned that they were interested to attend educational and communication activities about clonorchiasis.

**Fig 2:**
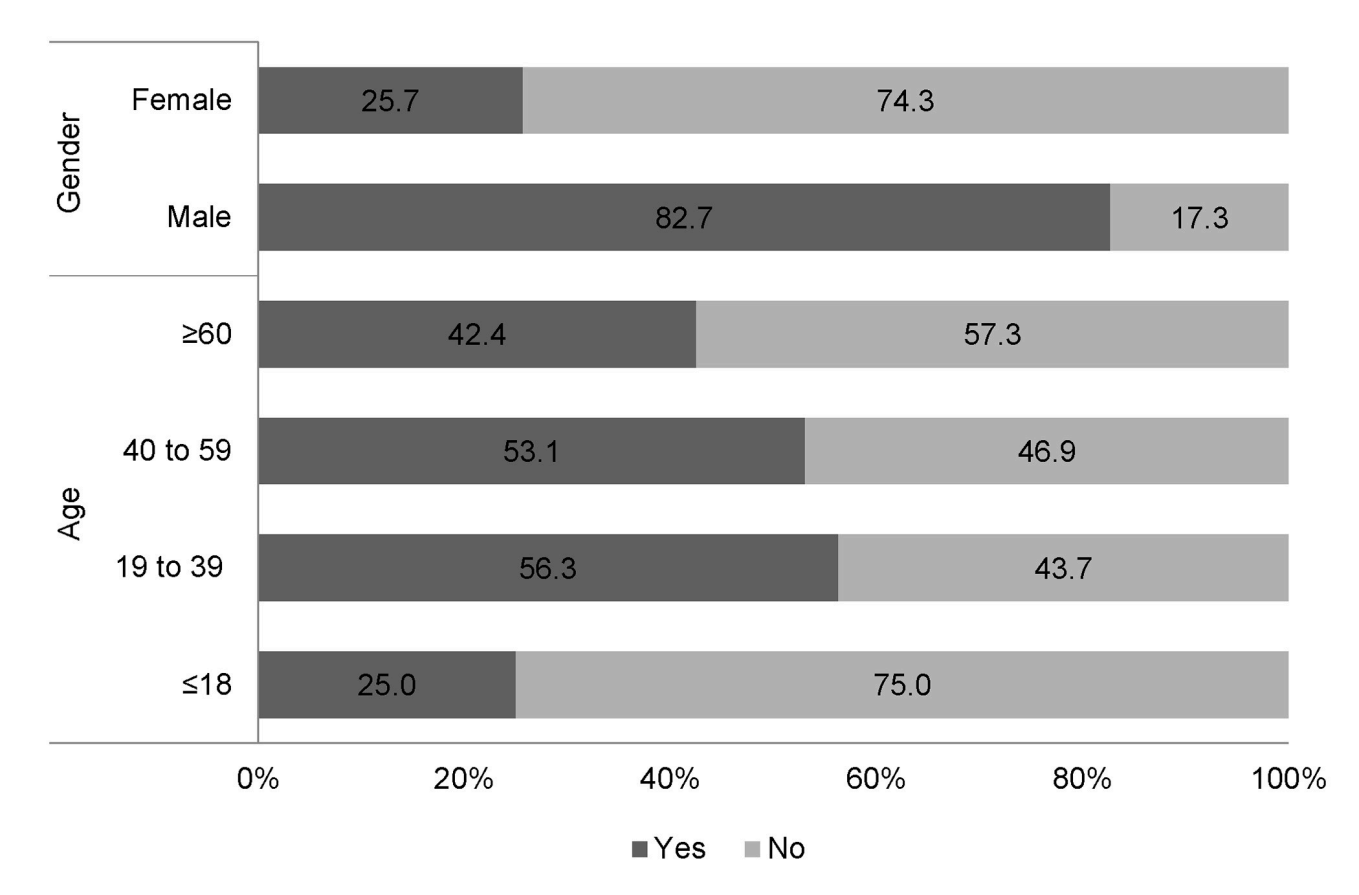
Differences in raw fish eating behavior by gender and age.

Past raw fish consumption (one or more than 1 year ago) was mentioned by 52.2% of participants, while 23% indicated that they had consumed a raw fish dish in the last year. Raw fish eating behavior was associated with gender and age group. Fig 2 shows that consumption of raw fish is much more common among males (82.7%) than in females (25.7%) (*p*<0.001). Individuals of the age group 19–39 years (56.3%) were more likely to eat raw fish than those of age group ≤ 18 (*p* = 0.007) and age group ≥ 60 (*p* = 0.026).

**Fig 3:**
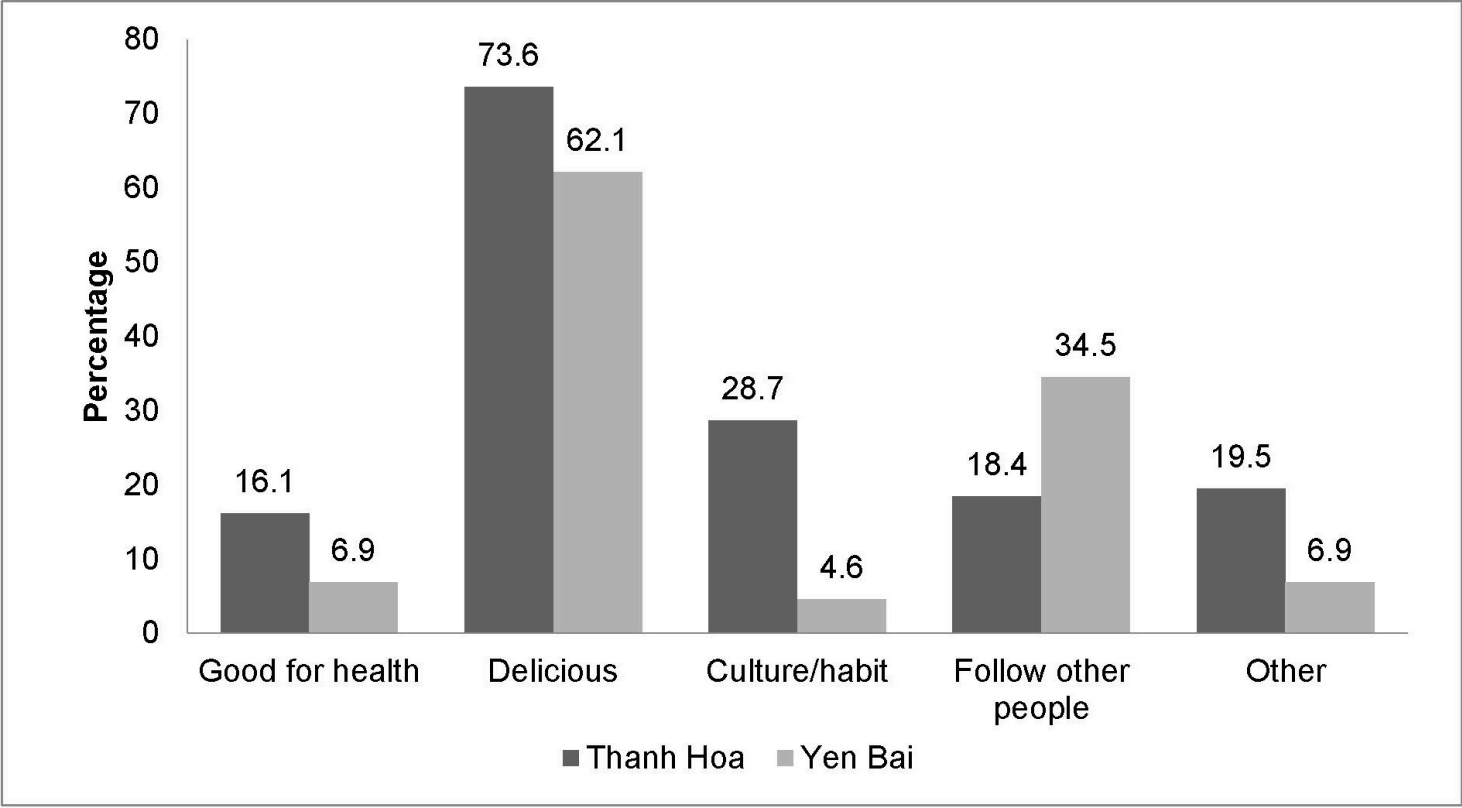
Reasons for people for keeping raw fish-eating behaviors during the last year.

Among the people who continued eating raw fish during the past year (n = 174), 67.8% declared to do so because they consider raw fish a delicacy (n = 118,), and 11,5% (n = 20) consider raw fish eating to be good for health. Other reasons mentioned were following the example of other people while eating (n = 46, 26.4%), it being part of their culture, or a habit (n = 29, 16.7%), with significant provincial differences (*p* = 0.001, *p* = 0.002) (Fig 3). While 97% of man drank alcohol while eating raw fish dishes, this was the case for only 28.1% of females.

**Fig 4:**
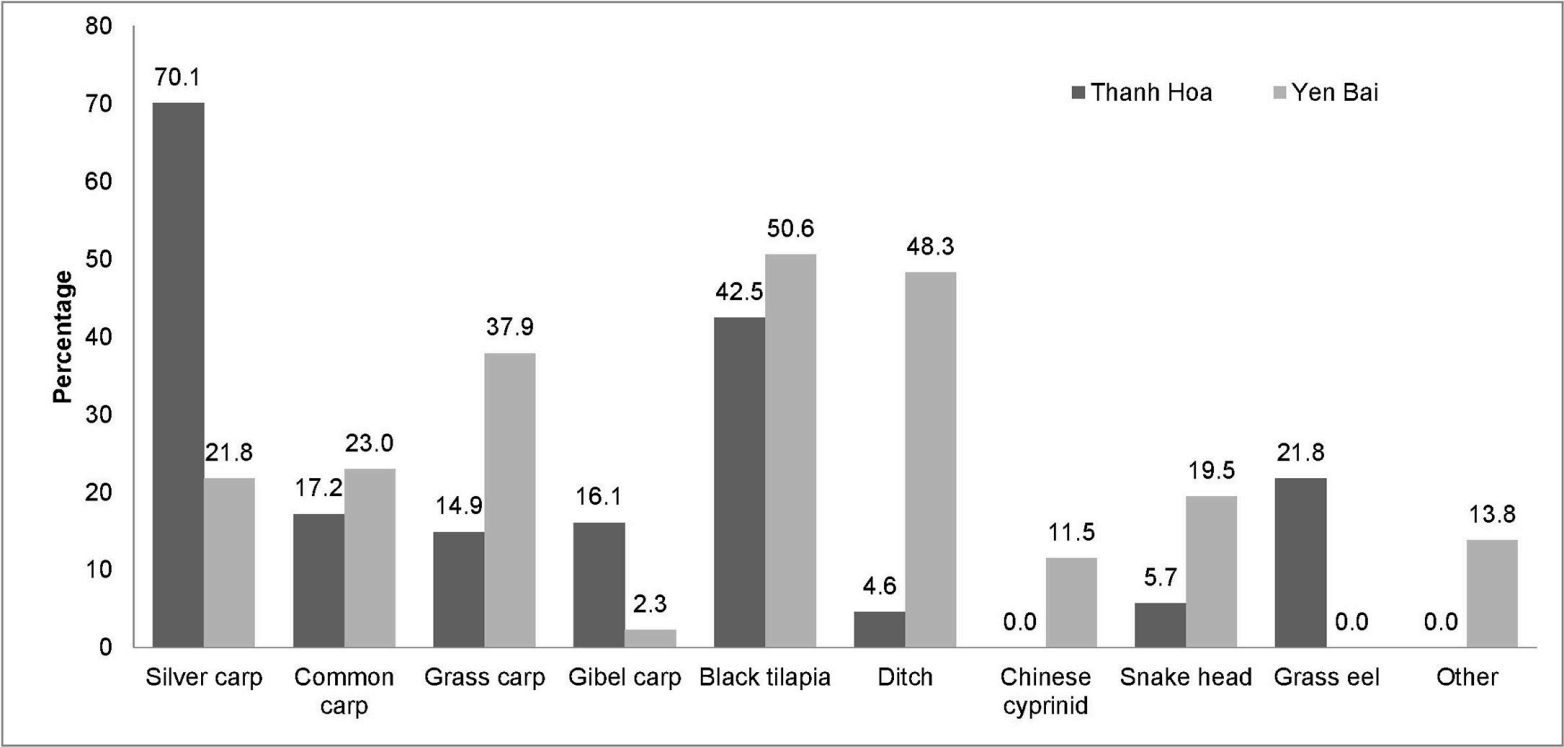
Fish species used for raw fish dish preparation (based on reports from people who ate raw fish in the last year).

Overall, the fish species most commonly consumed raw were black tilapia (*Oreochromis niloticus*) (46.6%) and silver carp (*Hypophthalmichthys molitrix*) (46%) (Fig 4), though there were significant differences in the fish species consumed raw between the two provinces (*p*< 0.010), except for the common carp (*Cyprinus carpio)*. For instance, Chinese cyprinid (*Toxabramis houdemeri*) was only consumed in Yen Bai province and grass eel (*Pisodonophis boro)* was only consumed in Thanh Hoa province.

**Fig 5:**
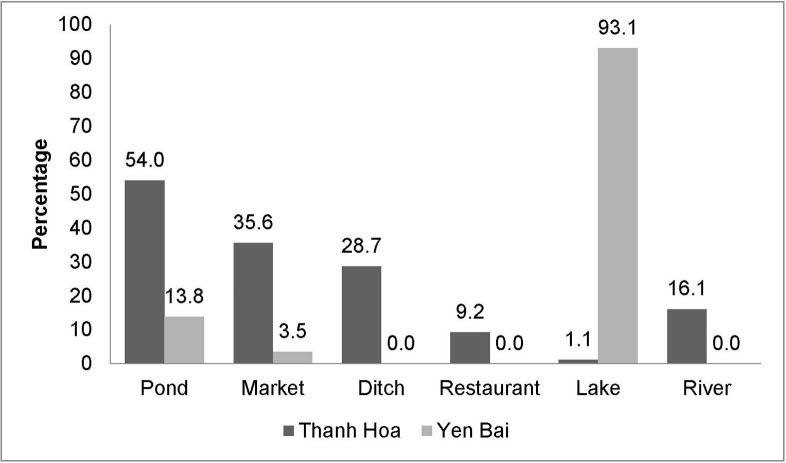
Origin of fish used for raw fish dish preparation (based on reports from people who ate raw fish in the last years).

There were significant differences in the source of fish used for raw fish dishes between the two provinces (*p*<0.010). In Yen Bai province, consumed raw fish mainly originated from Thac Ba Lake (93.1%). In Thanh Hoa province, the origin of the fish was more diverse: pond (54.0%), market (35.6%), river (16.1%), ditch (28.7%) were the most important sources (Fig 5).

Furthermore, more than half (57%) of participants reported that their families use the same knife or/and cutting board for preparing raw and cooked food dishes. In Thanh Hoa province, 43.9% of participants have a pond at home, while in Yen Bai province, only 19.8% have a pond. Among the 242 participants who have a pond, 28.5% revealed that they use human and animal feces to feed fish. In Yen Bai province, 8.4% of participants do not have a toilet at their house, they reported to defecate in fields/ditch, hills, and the garden and also in the Thac Ba lake. Another 8.3% of people in this province who have a pour flush toilet, said that the waste is discarded to streams, canals and rice fields or directly to the Thac Ba lake. In Thanh Hoa province, 4.9% of participants discharged the waste of the pour flush toilet in to their pond or in the neighbors’ pond.

### Prevalence of *C. sinensis*

Overall, 340 participants (40.4%) were found to be positive for eggs of *C*. *sinensis*. Stratifying *C*. *sinensis* positivity by commune, Phuc An (53.3%) was found to have the highest prevalence, followed by Ha Vinh (41.1%), Vu Linh (36%) and Ha Duong (26.5%). The positivity rate was higher in male (54.2%) than in female (28.3%), and highest in the age group between 19 to 39 years old (50.6%).

### Risk factors for *C. sinensis* infection

#### Univariable models

Based on the univariable models, gender, age, education, occupation, level of knowledge on clonorchiasis, history of eating raw fish, and type of toilet were all significantly associated with *C*. *sinensis* infection (*p*< 0.05). Male gender, low education, labor age group (19 to 39 and 40 to 59 years old), low level of knowledge on clonorchiasis, eating raw fish in the past (last one year or before), and using an unhygienic toilet, were risk factors for infection with *C*. *sinensis* (Table 2). The odds for infection were higher in mason and fisherman, and lower in students and traders, as compared to farmers. In contrast, factors such as, history of *C*. *sinensis* examination, history of *C*. *sinensis* treatment, perceived seriousness of *C*. *sinensis*, health concern about *C*. *sinensis* infection, habit of using knife and cut-board, were not significantly associated with a *C*. *sinensis* infection.

**Table 2:** Potential risk factors for *C. sinensis* infection in 4 communes in Yen Bai and Thanh Hoa provinces (univariable models).

Category	Total	% pos	OR	(95% CI)	P-value	AOR*	(95% CI)	P-value
**Commune**								
Ha Duong	185	26.5	1	reference				
Ha Vinh	219	41.1	1.94	1.27–2.96	0.002			
Phuc An	226	53.3	3.44	2.26–5.22	<0.001			
Vu Linh	211	36.0	1.56	1.02–2.40	0.042	** **	** **	** **
** Gender**								
Female	446	28.3	1	reference		1	reference	
Male	395	54.2	3.00	2.26–4.00	<0.001	3.4	2.52–4.58	<0.001
** Age**								
≤18	76	15.8	1	reference		1	reference	
19 to 39	253	50.6	5.46	2.81–10.61	<0.001	4.99	2.54–9.82	<0.001
40 to 59	424	42.0	3.86	2.02–7.36	<0.001	4.18	2.15–8.13	<0.001
≥60	88	25.0	1.78	0.81–3.89	0.150	2.16	0.97–4.84	0.061
** Education**								
Vocational & bachelor	53	18.9	1	reference		1	reference	
High school	145	37.9	2.63	1.22–5.65	0.013	2.7	1.24–5.87	0.012
Secondary school	331	40.8	2.96	1.44–6.10	0.003	2.99	1.44–6.22	0.003
Primary school or no education	228	52.2	4.69	2.25–9.80	<0.001	4.18	1.98–8.81	<0.001
** Occupation**								
Famer	430	43.7	1	reference		1	reference	
Government service & worker	107	34.6	0.68	0.44–1.06	0.087	0.77	0.49–1.21	0.261
Self-employed	143	42.7	0.96	0.65–1.40	0.824	0.91	0.62–1.34	0.627
Student	19	10.5	0.15	0.03–0.66	0.012	0.19	0.04–0.87	0.032
Trade	28	25.0	0.42	0.18–1.03	0.058	0.40	0.16–0.96	0.040
Mason & fisherman	30	80.0	5.15	2.06–12.85	<0.001	5.10	2.0–12.89	0.001
** Knowledge level**								
High	93	34.4	1	reference		1	reference	
Medium	380	43.7	1.48	0.92–2.37	0.105	1.52	0.93–2.45	0.092
Low	284	42.6	1.42	0.87–2.31	0.163	1.67	1.01–2.76	0.045
** Level of seriousness**								
Not serious	139	38.9	1	reference		1	reference	
Somewhat serious	40	32.5	0.76	0.36–1.60	0.465	0.7	0.33–1.49	0.358
Serious	295	44.1	1.24	0.82–1.87	0.305	1.09	0.71–1.66	0.699
Very serious	283	43.1	1.19	0.79–1.81	0.404	0.94	0.61–1.44	0.763
**Level of concern**								
No concern	152	38.8	1	reference		1	reference	
A little concern	245	46.1	1.35	0.89–2.07	0.154	1.15	0.75–1.76	0.519
Concern	360	40.8	1.09	0.74–1.60	0.671	0.88	0.59–1.32	0.531
** History of eating raw fish**								
Never	362	20.2	1	reference		1	reference	
Yes, before 1 year ago	221	54.8	4.79	3.31–6.93	<0.001	4.56	3.13–6.63	<0.001
Yes, during last year	174	71.8	10.10	6.65–15.35	<0.001	10.03	6.56–15.34	<0.001
** Clonorchiasis examination history**								
Yes	218	43.6	1	reference		1	reference	
No	539	41.6	0.92	0.67–1.27	0.610	1.04	0.74–1.46	0.824
** Clonorchiasis treatment history**								
Yes	132	51.5	1	reference		1	reference	
No	625	40.2	0.63	0.43–0.92	0.017	0.72	0.49–1.06	0.099
** Habit of using cutting board and knife**								
Same knife and/or cutting board	432	44.2	1	reference		1	reference	
Separate knife & cutting board	325	39.4	0.82	0.61–1.10	0.183	0.76	0.56–1.03	0.076
** Toilet type**								
Hygienic	647	38.8	1	reference		1	reference	
Unhygienic	78	65.4	2.98	1.82–4.88	<0.001	2.89	1.73–4.80	<0.001
No toilet	32	53.1	1.79	0.88–3.64	0.110	1.58	0.75–3.31	0.227

#### Multivariable analysis

In the final multivariable model, gender, age, history of eating raw fish, type of toilet use, and history of clonorchiasis treatment were retained as predictors, and they were all significantly associated with *C*. *sinensis* infection. The odds of infection with *C*. *sinensis* was 2.00 (95%CI: 1.31–3.05, *p* = 0.001) times higher in males than in females (Table 3). Individuals belonging to the 19 to 39 years age groups had a 6.46 (95% CI: 1.25–33.37, *p* = 0.026) times higher odds of infection than those under 19 years old (Table 3). Participants with a lower educational level had a higher odds for *C*. *sinensis* infection than those with a higher educational level. Eating raw fish was the most important risk factor, especially for people who ate raw fish during the last year, they had odds of being infected with *C*. *sinensis* 8.00 (95% CI: 4.78–13.36, *p*<0.001) times higher as compared to those who declared that they had never eaten raw fish (Table 3). Participants living in a household using an unhygienic toilet had a 2.74 (95% CI: 1.53–4.29, *p* = 0.001) times higher odds of infection than those who use a hygienic toilet. People who did not get a clonorchiasis treatment had a higher risk to be infected with *C*. *sinensis* than people who had received the treatment. People in Phuc An commune, Yen Bai province, had a higher odds for *C*. *sinensis* infection than people who lived in Ha Duong commune, Thanh Hoa province (Table 3).

**Table 3:** Risk factors for *C. sinensis* infection in 4 communes in Yen Bai and Thanh Hoa provinces (multivariable model) (n = 757).

Category	Total	% pos	AOR	(95% CI)	P-value
**Commune **					
Ha Duong	165	29.09	1	reference	
Ha Vinh	218	40.83	1.54	0.92–2.59	0.100
Phuc An	195	56.41	2.38	1.38–4.10	0.002
Vu Linh	179	40.22	1.37	0.79–2.39	0.260
**Gender**					
Female	405	28.64	1	reference	
Male	352	57.67	2.00	1.31–3.05	0.001
** Age**					
≤18	20	10.00	1	reference	
19 to 39	245	49.80	6.46	1.25–33.37	0.026
40 to 59	407	42.75	4.64	0.91–23.77	0.066
≥60	85	24.71	2.76	0.50–15.14	0.244
** Education**					
Vocational & bachelor	53	18.90	1	reference	
High school	145	37.93	2.88	1.22–6.79	0.016
Secondary school	331	40.79	3.92	1.73–8.87	0.001
Primary school or no education	228	52.19	5.57	2.37–13.07	<0.001
** History of eating raw fish**					
Never	362	20.17	1	reference	
Yes, before 1 year ago	221	54.75	3.82	2.43–6.01	<0.001
Yes, during the last year	174	71.84	8.00	4.78–13.36	<0.001
** Clonorchiasis treatment history**					
Yes	132	51.52	1	reference	
No	625	40.16	1.82	1.15–2.89	0.011
** Toilet type**					
Hygienic	647	38.79	1	reference	
Unhygienic	78	65.38	2.74	1.53–4.92	0.001
No toilet	32	53.13	1.80	0.76–4.27	0.184

## Discussion

Our findings demonstrated that *C*. *sinensis* infection is widespread both in Yen Bai and Thanh Hoa provinces in Vietnam. The overall prevalence estimates (40.4%), was higher than those earlier reported in Vietnam [17], which might be due to easier access to wild caught and freshwater fish in the current study as compared to other study areas, or higher infection rates in fish in the area. Moreover, up to 52% of participants indicated that they had eaten raw fish dishes, thus putting themselves at risk of infection. Indeed, according to the results in the present study, the habit of eating raw fish in the last year increased the risk of *C*. *sinensis* infection by 8 times. Raw fish consumption thus represents the key risk factor for *C*. *sinensis* infection, as was demonstrated in other studies in Vietnam (and other Asian countries) [5, 19–21, 29], but was now confirmed in a multivariable model. Interestingly, a high proportion of participants (20.7%) who declared to have never consumed raw fish were also infected with *C*. *sinensis*. This finding could be explained by recall or emotional bias for raw fish consumption (i.e having forgotten past consumption, or feeling ashamed to admit). An alternatively hypothesis might be the cross-contamination of food by kitchen utensils by metacercariae as reported in other studies [19–21, 29, 30], supported by the fact that a large number of people (57%) in this study used the same knife or cutting board for preparing raw and cooked food dishes.

We identified some regional differences in reasons for raw fish consumption. Participants in Thanh Hoa province predominantly declared that they eat raw fish because of cultural traditions, while those in Yen Bai province indicated to mainly mirror the behavior of others. Thus in both regions, eating raw fish is a deeply rooted habit influenced by social factors. Convincing people to give up such a behaviour is challenging as was demonstrated in Thailand, where a 10-year intensive opisthorchiasis control program led to a decline in regular raw fish consumption (14% to 7%), yet occasional raw fish consumption remained rampant [31].

In this study, the fish species reported as being used to prepare raw fish dishes are known to be infection sources of *C*. *sinensis* in Vietnam [24, 32–35]. A significant association was observed between the source of raw fish dishes and province. The main source of fresh water fish in Yen Bai province is the Thac Ba Lake, while in Thanh Hoa, the main sources of caught fish are pond, market, field/canal and river. Interestingly, toilet type was also significantly associated with province, and with *C*. *sinensis* infection. The habit of directly defecating in fields, hills, and garden and also in the Thac Ba lake or use of unhygienic toilets such as pour flush toilet, allow the maintenance of the life cycle of the liver fluke. In addition, natural disasters such as yearly flooding in Thanh Hoa, can also lead to the dissemination of snails and fish.

We found a clear relationship between gender and *C*. *sinensis* infection, as well as between gender and raw fish consumption. Differences in prevalence of *C*. *sinensis* by gender have been described elsewhere in the region [2, 3, 6, 36], including in Vietnam [18–20, 30]. Consumption of raw fish in Vietnam is often associated with social gatherings, to which mainly men participate and during which alcohol use is high [22]. Nevertheless, our results showed a significant difference in infection rates with *C*. *sinensis* between males (54.2%) and females (28.3%), even when adjusting for raw consumption habits.

In our study, age was significantly associated with *C*. *sinensis* infection, with the prevalence of *C*. *sinensis* being the highest in the age group 19–39 years, contrasting results from earlier studies conducted in Ninh Binh province and Thanh Hoa province, where the highest prevalence was observed in the age group 40–49 years [18, 19]. This difference may be the result of raw fish consumption starting at a younger age in the areas that we surveyed, or other unidentified, age-specific factors.

People with a low level of education had a high risk of *C*. *sinensis* infection. A similar finding was observed in Ninh Binh province [20]. Moreover, 17.3% of respondents had never heard about liver fluke, a higher percentage than earlier observed in Thanh Hoa province [18] (6.3%), but lower than estimated in Ninh Binh province [20] (69.0%). Although the knowledge score was not retained in the final multivariable model, health education campaigns should be implemented to raise awareness on risk factors and disease prevention, especially as a significant proportion of the population has misconceptions about disease transmission and prevention. For instance, lemon and alcohol were mentioned as effective approach to kill the parasite. Furthermore, feeding fish with human and animal feces is still common practice in the area, and even considered an appropriate way to ensure a good nutritional status of fish, even though this practice will maintain the life cycle of *C*. *sinensis*. In northern Vietnam the use of animal feces in fish ponds is practiced within the context of the agricultural VAC system, an acronym for “*Vuon*”, “*Ao*”, “*Chuong*”, which means garden, fish pond and animal shed, respectively. The implementation of this VAC system has been encouraged by the Vietnamese government, as it is considered an ecological approach for fish breeding and is shown to have positive effects on the financial, health and nutritional status of the farmers [32, 37–39]. Nevertheless, such practices facilitate the transmission of fish-borne zoonotic trematodes.

One of the limitations of our study is that the diagnostic procedure may have overestimated the true prevalence. Indeed, distinguishing eggs of *C*. *sinensis* with those of minute intestinal flukes based on microscopy remains a challenge [40]. Nevertheless, differentiating parasite eggs obtained by the FECT is considerably more accurate as compared to Kato-Katz, because it allows the demonstration of the characteristic prominent wrinkling on the egg shell surface, and the prominence of the operculum and shoulder rims, and the terminal protuberance [27, 28].

In conclusion, we have demonstrated that clonorchiasis is highly endemic in communes near Thac Ba lake–the mountainous areas in Yen Bai province, and in delta regions in Thanh Hoa province. The main factors associated with infection in these endemic areas are male gender, consumption of raw fish, low level of education and use of unhygienic toilets. Moreover, lack of knowledge of *C*. *sinensis* infection leads to bad practices in food consumption and hygiene. Our study showed that 20.2% of participants declaring not to eat raw fish, were infected, thus more studies should be conducted on the risk of transmission during food preparation in these endemic areas. More epidemiological studies are needed to have a comprehensive overview of the disease in the wider region as well as to evaluate the effectiveness of intervention strategies including mass administration of anti-helmintic drugs and educational campaigns to raise awareness in high endemic areas. Moreover, socio-anthropological research is vital to improve our understanding of the drivers of the cultural habits and assess the acceptability of educational messages and interventions.

## Supporting information

S1 Checklist STROBE checklist cross-sectional studies. (DOC)

S1 Table Knowledge questions. (XLSX)
